# Biological and Clinical Consequences of Integrin Binding via a Rogue RGD Motif in the SARS CoV-2 Spike Protein

**DOI:** 10.3390/v13020146

**Published:** 2021-01-20

**Authors:** Lee Makowski, William Olson-Sidford, John W. Weisel

**Affiliations:** 1 Department of Bioengineering, Northeastern University, Boston, MA 02445, USA; olson-sidford.w@northeastern.edu; 2 Department of Chemistry and Chemical Biology, Northeastern University, Boston, MA 02445, USA; 3 Department of Cell and Developmental Biology, University of Pennsylvania, Philadelphia, PA 19104, USA; weisel@pennmedicine.upenn.edu

**Keywords:** COVID-19, spike protein, receptor-binding domain, RGD motif, integrins, coagulation, angiogenesis

## Abstract

Although ACE2 (angiotensin converting enzyme 2) is considered the primary receptor for CoV-2 cell entry, recent reports suggest that alternative pathways may contribute. This paper considers the hypothesis that viral binding to cell-surface integrins may contribute to the high infectivity and widespread extra-pulmonary impacts of the SARS-CoV-2 virus. This potential is suggested on the basis of the emergence of an RGD (arginine-glycine-aspartate) sequence in the receptor-binding domain of the spike protein. RGD is a motif commonly used by viruses to bind cell-surface integrins. Numerous signaling pathways are mediated by integrins and virion binding could lead to dysregulation of these pathways, with consequent tissue damage. Integrins on the surfaces of pneumocytes, endothelial cells and platelets may be vulnerable to CoV-2 virion binding. For instance, binding of intact virions to integrins on alveolar cells could enhance viral entry. Binding of virions to integrins on endothelial cells could activate angiogenic cell signaling pathways; dysregulate integrin-mediated signaling pathways controlling developmental processes; and precipitate endothelial activation to initiate blood clotting. Such a procoagulant state, perhaps together with enhancement of platelet aggregation through virions binding to integrins on platelets, could amplify the production of microthrombi that pose the threat of pulmonary thrombosis and embolism, strokes and other thrombotic consequences. The susceptibility of different tissues to virion–integrin interactions may be modulated by a host of factors, including the conformation of relevant integrins and the impact of the tissue microenvironment on spike protein conformation. Patient-specific differences in these factors may contribute to the high variability of clinical presentation. There is danger that the emergence of receptor-binding domain mutations that increase infectivity may also enhance access of the RGD motif for integrin binding, resulting in viral strains with ACE2 independent routes of cell entry and novel integrin-mediated biological and clinical impacts. The highly infectious variant, B.1.1.7 (or VUI 202012/01), includes a receptor-binding domain amino acid replacement, N501Y, that could potentially provide the RGD motif with enhanced access to cell-surface integrins, with consequent clinical impacts.

## 1. Introduction

The clinical trajectory of COVID-19 disease, although widely variable, can be divided into an early, non-severe phase with upper airway involvement and, in some patients, a second, severe and potentially lethal phase with high viral load, hypoxia, “ground glass” infiltrates in the lung, widely disseminated coagulopathy, pathological pulmonary angiogenesis and progression to acute respiratory distress syndrome [1,2]. The second phase, seen in patients with severe disease, appears to be driven by a strong inflammatory response, with upregulation of cytokine and interferon production and an associated cytokine storm [2,3,4,5]. Acute disease may be accompanied by myocardial dysfunction and arrhythmia, acute coronary syndromes, acute kidney injury, gastrointestinal symptoms, hepatocellular injury, hyperglycemia and ketosis, neurologic illnesses, ocular symptoms and dermatologic complications [1]. Global efforts are underway to identify the attributes of the SARS CoV-2 virus that underpin this unique clinical presentation as a step toward identifying potential therapeutic strategies.

ACE2 (angiotensin converting enzyme 2) was identified early as the primary receptor for CoV-2 binding and cell entry, and its high affinity to CoV-2 spike protein (compared to SARS CoV-1) was implicated in the far greater infectivity of SARS CoV-2. More recently, mapping of the tissue distribution of ACE2 expression and evidence of additional binding interactions between virion and cell surface receptors have suggested that the situation may be more complex. Reports have indicated that SARS-CoV-2 virion binds neutropilin-1 [6] and CD147 [7], two cell-surface receptors that could facilitate viral entry. Sigrist et al. [8] argue that the presence of an RGD (arginine-glycine-aspartate) motif in the spike protein suggests that integrins may provide an alternate cell entry pathway. Interactions between the spike protein and integrin α_5_β_1_ have also been reported [9] but require further confirmation.

Early reports of strong expression of ACE2 in the upper airway epithelium [10,11] and its abundant presence in the epithelia of the lung and small intestine [12], in the vascular endothelium [11] and in specific respiratory, corneal and intestinal epithelial cells were consistent with ACE2 being the most likely path for cell entry for SARS-CoV-2. However, recent reports have contradicted those studies: Hikmet et al. [13] reported that ACE2 expression in the respiratory tract is limited compared to that in other barrier tissues and observed only very low levels of ACE2 in the normal respiratory system. Other investigators observed viral RNA in ciliated but not secretory epithelial cells in the airways of COVID-19 patients (Hou et al., 2020). These contradictory observations were apparently resolved by the demonstration that ACE2 expression is induced by interferons [14]. In particular, viral infection of the upper respiratory tract could trigger a rapid interferon-induced increase in ACE2 expression in lower airways and lung parenchyma, providing a route for viral spread across the respiratory mucosa [15]. Nevertheless, the level of ACE2 expression in respiratory tissue is sufficiently low as to motivate the examination of additional routes for viral entry.

This paper considers the hypothesis that integrins may provide an alternate path for SARS CoV-2 cell entry and that the frequently observed coagulopathy, angiogenesis and other clinical impacts observed during the severe second phase of COVID-19 infection are driven not only by the widely reported inflammatory response but, at least in part, by the direct interaction of virus particles with endothelial cells and platelets, among others. Coagulation and angiogenesis are cellular processes regulated, to some degree, by integrins. Evidence is considered here that suggests that an integrin-binding “RGD motif” near the distal tip of the SARS CoV-2 spike protein may interact with integrins on cell surfaces and lead to extensive dysregulation of these processes.

Early in infection, SARS-CoV-2 targets nasal and bronchial epithelial cells and pneumocytes [2]. During this stage, integrins may enhance viral entry either by acting as co-receptors or alternate pathways for cell entry. At this stage of the disease, symptoms are largely respiratory and integrins relevant to coagulation and angiogenesis are sequestered from interactions with virions. In later stages, viral replication accelerates [16] and the epithelial–endothelial barrier is compromised [2], leading to viremia in roughly 15% of patients [17]. Virions then gain access to the vasculature and to cell-surface receptors on endothelial cells, platelets and eventually other diverse cell types. This may open the flood gates to the widely diverse pathological impacts observed in the second phase of disease.

If integrins provide SARS CoV-2 with an ACE2-independent route to cell entry, the key interaction will be with the RGD motif in the receptor binding domain of the spike protein. The RGD (arg-gly-asp) tri-peptide motif is recognized by at least eight human integrins and binds to their extracellular domains with high affinity. Many human viruses use an RGD motif displayed on their virion surface to bind to the extracellular domain of integrins, thereby initiating cell entry and triggering dysregulated cellular signaling processes [18,19]. For instance, binding to integrin α_IIb_β_3_, the principal platelet integrin, could dysregulate platelet aggregation. Initially promulgated through binding to the KQAGDV sequence in the γC region of fibrinogen, aggregation later involves binding of RGD sequences in the α chain of fibrin to integrin α_IIb_β_3_. Similarly, virion binding to integrin α_V_β_3_ could lead to dysregulation of angiogenesis since integrin α_V_β_3_ is one of at least six RGD-binding integrins implicated in the regulation of vascular growth [20,21]. In the absence of experimental data probing the affinity of the SARS CoV-2 spike protein to relevant human integrins, this paper explores the possibility that these interactions occur and are responsible for important clinical aspects of COVID-19.

### 1.1. Rationale

Suspicion that the spike protein RGD motif might contribute to COVID-19 pathology is based on both clinical and molecular considerations:

#### 1.1.1. Clinical

SARS-CoV-2 is more highly infectious than SARS-CoV-1, which may in part be explained by higher affinity binding to the ACE2 receptor, but the addition of an alternate cell entry pathway provided by binding to integrins would greatly increase the probability and strength of viral interactions with airway cells. Examination of the lungs of COVID-19 victims at autopsy indicates that they exhibit unusually high levels of microthrombi (9× that of comparable influenza victims) and aberrant angiogenesis (2.7× that seen in influenza) [3]. Unchecked immune responses are unlikely to provide the full explanation for the systemic coagulopathy and thromboembolic complications observed in the majority of severe cases [4,22]. Intussusceptive angiogenesis constitutes an unexpected aspect of lung tissue disruption frequently observed in COVID-19 victims at autopsy [3]. Both thrombosis and angiogenesis are regulated, at least in part, by integrin-mediated binding interactions and signaling, raising suspicion about the possible involvement of integrin-binding agents in COVID-19 pathology.

#### 1.1.2. Spike Protein Structure

The spike protein of SARS CoV-2 virus contains an RGD motif near the distal tip of its receptor-binding domain [23] with structural features reminiscent of known integrin-binding proteins. Direct measurements of the potential binding of spike protein to relevant integrins have yet to be reported. In the absence of these critical data, this paper examines the structure of the RGD motif in the SARS CoV-2 spike protein and compares it with structures of RGD motifs on known integrin ligands and integrin-targeting virions. Is the structure of SARS CoV-2 spike protein sufficiently similar to that of known integrin-binders to support the possibility that the SARS CoV-2 spike protein is capable of binding to integrins? What interactions between the spike protein RGD motif and integrins would be required to generate the observed pathological coagulation? Is it possible that direct interaction of virion and integrins could be responsible for abnormal angiogenesis observed in COVID-19? Could SARS CoV-2 virion interactions with integrins contribute to the very high infectivity of CoV-2?

## 2. Structure of the RGD Motif on the Spike Protein of SARS CoV-2

**Figure 1:**
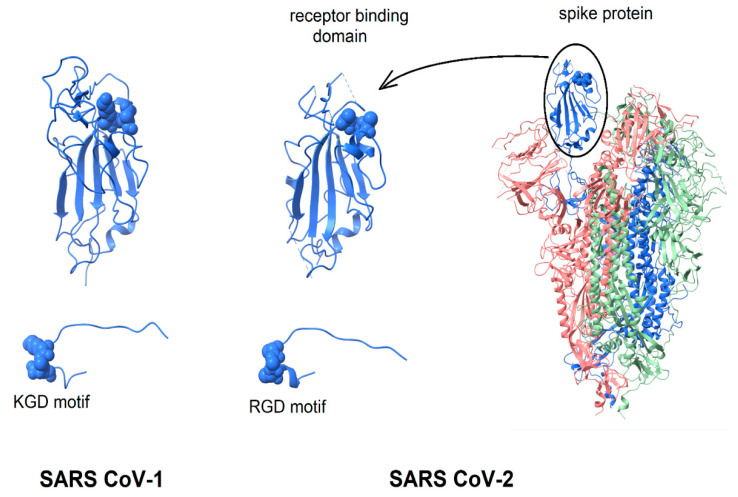
Display of integrin-binding motifs on coronavirus spike proteins. In each image, the integrin-binding motif is rendered in “space filling mode” and the remainder of the protein in ribbons. SARS CoV-1 (left) contains a KGD motif; SARS CoV-2 an RGD motif. Left to right: SARS CoV-1 receptor-binding domain (6acd); SARS CoV-2 receptor-binding domain (6vsb); SARS CoV-2 spike protein (6vsb). The three chains of the trimer are colored blue, green and pink. The RGD/KGD motif is at or near the protein surface. Adjacent surface loops would need to move to allow binding to an integrin. The protein segment containing the RGD/KGD motif has been dissected from the remainder of the protein and enlarged to show that in both SARS CoV-1 and SARS Cov-2 spike proteins, the RGD/KGD motif is at a tight bend where the peptide chain reverses direction, similar to known integrin-binding molecules.

Given the extensive evidence of virion-displayed RGD motifs binding to integrins in a functionally relevant manner, the question arises, “Is the structure of the SARS CoV-2 spike protein consistent with the possibility that its RGD-motif could bind to integrins?” This question is considered by the comparison of the spike protein structure with that of known integrin-binding proteins. As shown in Figure 1, in the intact spike protein, the RGD motif is located at the distal tip of the protein, on the surface of the receptor-binding domain, forming a bend where the direction of the peptide chain reverses [23,24]. Surrounded by protein segments that are highly flexible, it appears sufficiently exposed to solvent that only minor conformational changes of flexible protein segments would be required to enable integrin-binding (see below). In Figure 1, the spike protein is shown in a configuration with one receptor-binding domain in an open, active conformation and the other two in a closed conformation [24]. The spike protein of SARS CoV-1 is very similar in structure and is included in Figure 1 for comparison.

**Figure 2:**
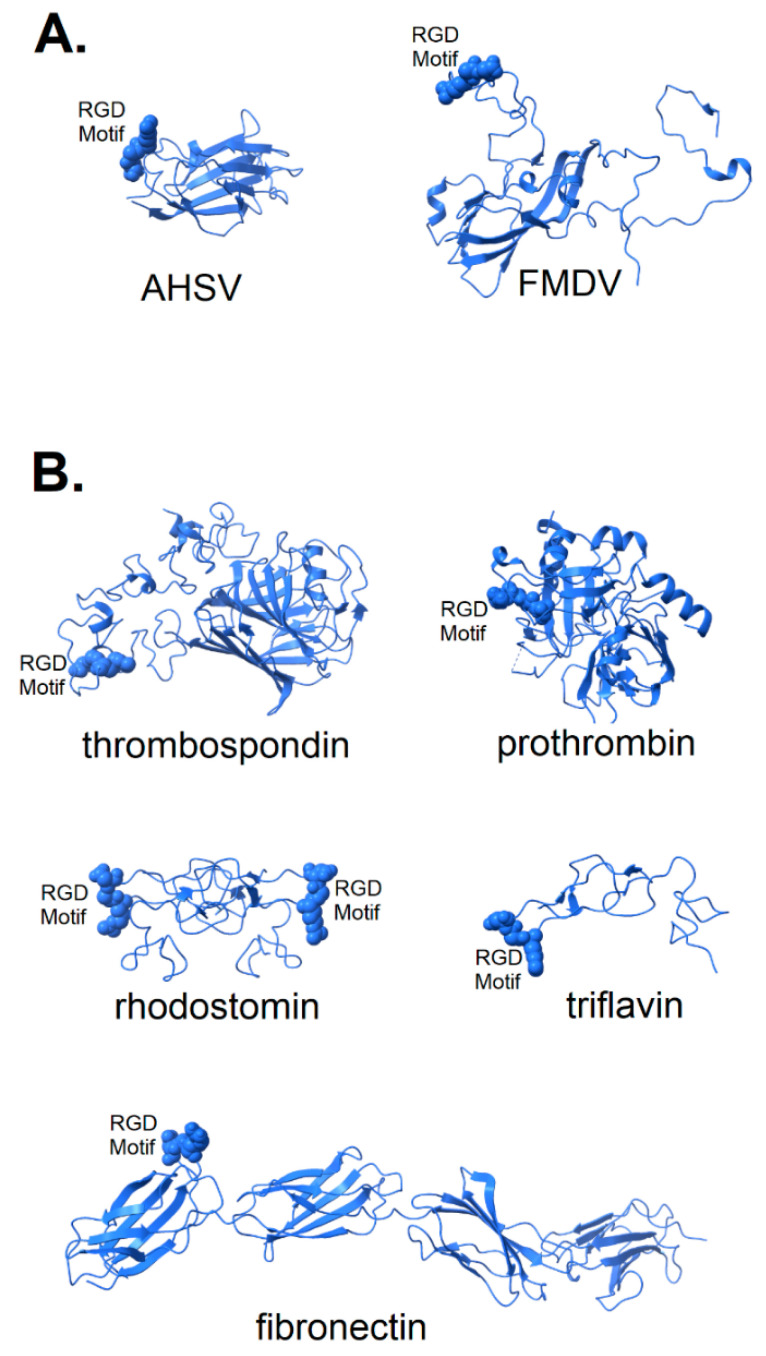
Structure of known integrin-binding proteins: (**A**) Virion proteins known to bind integrins through an RGD motif (shown in space-fill) include (right) foot and mouth disease virus capsid protein (5neu—this RGD motif is highly flexible prior to integrin-binding, but structurally stabilized when bound to integrin—image is from a co-crystal of the capsid protein and integrin with integrin structure removed to make visible the RGD domain); (left) African horse sickness virus (1ahs—top domain of capsid protein VP7). (**B**) Other proteins known to bind integrin through an RGD motif: thrombospondin (1ux6); prothrombin (3u69); rhodostomin (4rqg) and triflavin (1j2l) are disintegrins, small toxins from snake venom with high affinity to integrins; and fibronectin (1fnf—domains 6–10)—an extracellular matrix protein with an integrin-binding RGD motif in its 10th domain.

In Figure 2, the structures of a number of integrin-binding viral proteins and natural integrin ligands are shown for comparison with the spike protein of SARS CoV-2. The coat protein of foot and mouth disease virus (FMDV) is shown in its integrin-bound conformation (pdb file 5neu), with integrin removed for clarity. Prior to binding, the FMDV RGD motif is part of a long, flexible loop [25,26] that is largely immobilized by binding to integrin. Also shown in Figure 2A is the top domain of capsid protein VP7 of the African horse sickness virus. This virion displays its RGD motif prominently near one end of the protein in a manner that would provide ready access to integrin binding sites (pdb file 1ahs).

The structure of two disintegrins, rhodostomin (4rqg) and triflavin (1j2l), small protein components of viper venom that bind tightly to integrin α_IIb_β_3_ on platelets, can be seen in Figure 2B to have RGD motifs that extend from the body of the protein to facilitate interaction with integrins. These toxic proteins act as potent anti-coagulant drugs by blocking fibrinogen-binding to integrin α_IIb_β_3_. Naturally occurring integrin ligands are also depicted in Figure 2B, including thrombospondin (1ux6), prothrombin (3u69) and fibronectin (1fnf), all exhibiting RGD motifs that extend from the body of the protein. Generally, the RGD motif of naturally occurring integrin ligands is either highly exposed on the protein surface, highly flexible or both. This would suggest that efficient binding of a spike protein to an integrin could be dependent on a microenvironment conducive to the disordering of the extreme distal tip of the spike protein. Small variation in microenvironmental conditions could lead to large fluctuations in the availability of the RGD motif for integrin-binding and lead to consequent variability in clinical manifestations.

### 2.1. Accessibility of the Spike Protein RGD Motif

**Figure 3:**
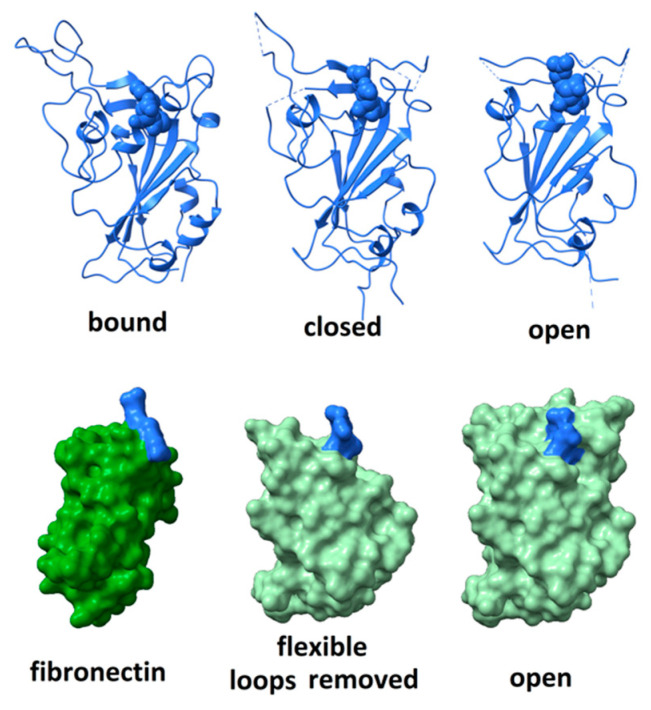
(top) Comparison of the structure of the receptor-binding domain when bound to ACE2 receptor (left—with ACE2 removed for clarity); (middle) unbound in the closed configuration; and (right) unbound in the open configuration. RGD motif rendered in space-fill. The distal surface loop undergoes dramatic rearrangements in different environments. (bottom) The receptor-binding domain of SARS CoV-2 spike protein in the “open” conformation (right and middle) compared to the structure of the integrin-binding domain 10 of fibronectin (left). Space-fill rendering to show the degree of exposure for the RGD domain when the flexible distal tip (residues 437 to 508) of the receptor-binding domain is removed (middle). The RGD motifs are indicated in blue.

Although the RGD motif in the SARS CoV-2 spike protein does not appear as free to move as seen in the FMDV coat protein, nor as exposed to solvent as in disintegrins or fibronectin, it is surrounded by highly flexible peptide segments that might fluctuate in a manner facilitating integrin-binding. The degree of flexibility can be observed by comparing, as in Figure 3 (top right), the structure of the receptor-binding domain in its open, unbound conformation (6vsb)—in which the positions of residues 444–448, 455–490, 501–502 are not defined due to molecular motions—(top middle) its structure in a closed, unbound conformation (6vsb); and (top left) its structure when bound to ACE2 receptor (6m0j—with ACE2 receptor removed for clarity). When bound to ACE2, the distal part of the receptor-binding domain is stabilized and all residues are visualized. The RGD motif comprises residues 403–405, shown in space-fill rendering in the figure. Comparison of these three structures indicates that dramatic rearrangement and ordering of the surface loops occurs on receptor-binding and suggests a degree of flexibility capable of providing significant solvent exposure to the RGD motif.

To assess how much flexibility is required to fully expose the RGD motif to solvent, amino acid residues proximal to the motif were computationally pruned until the entire motif was exposed to solvent. As shown in Figure 3 (bottom)**,** this requires the removal of the large surface loop comprising residues 437–508. Given the rearrangement that this loop undergoes on ACE2 receptor-binding, it seems plausible that, in certain microenvironments, it could fluctuate enough to allow the RGD motif to bind an integrin. Variability in microenvironmental factors that modulate either integrin or spike protein conformation may have a profound impact on the level of damage that viral invasion could wreak.

More compelling is the possibility that single amino acid substitutions may lead to a significant increase in the exposure of the RGD motif, enhancing the potential of spike protein-binding to integrins. The recurrent emergence of amino acid replacement N501Y, within the large surface loop, 437–508, has the potential for enhancing the flexibility of surface loops, thereby exposing the RGD motif to solvent in a way that is conducive to integrin-binding.

### 2.2. Integrin Specificity

The specificity of ligands for individual integrins is modulated by both the conformational environment and amino acid sequences immediately adjacent to the RGD motif, providing a layer of specificity required for the functioning of the integrin cell-regulatory network. Should conformational fluctuations of the CoV-2 spike protein expose the RGD motif enough to make possible binding to integrins, amino acids proximal to the RGD motif could modulate its binding to specific integrins. Comparison of the Cov-2 spike sequence with those of integrin-binding proteins provides little insight into possible integrin specificity. Not only do the RGD-proximate amino acids of spike protein appear to have little sequence similarity to integrin-binding proteins, but the corresponding sequences of integrin ligands appear to have relatively little similarity to one another. This is shown in Table 1, wherein the sequence of CoV-2 spike protein +/−15 residues from the RGD motif is aligned with sequences of SARS CoV-1 spike protein and 14 known integrin-binding proteins. It is difficult from these comparisons to discern a pattern that provides insight into integrin-binding specificity.

**Figure 4:**
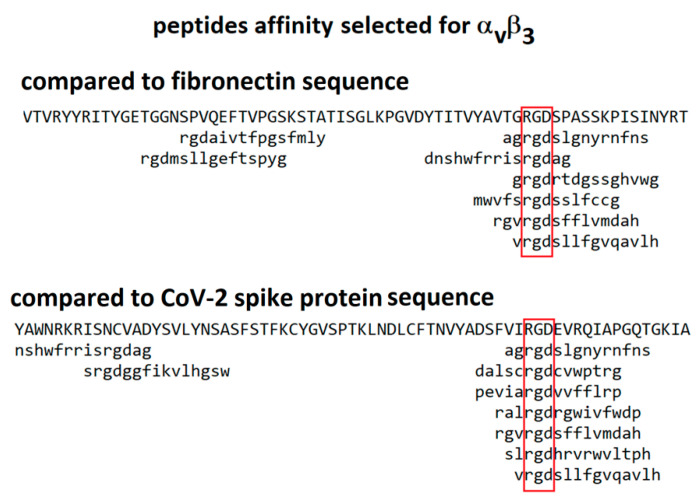
Alignment of peptides selected for affinity to integrin α_v_β_3_ against the sequences of fibronectin and CoV-2 spike protein. The red boxes indicate position of the ’rgd’ motif. The library screened was constructed of 15-mer linear peptides [27].

An alternative approach is to compare the sequence of the spike protein with those of peptides identified as binding to integrins by affinity selection from combinatorial phage-displayed libraries. During the 1990s, a number of publications reported the sequences of phage-displayed peptides selected for binding to integrins α_iib_β_3_, α_v_β_3_, α_v_β_5_ and α_1_β_5_ [27,28,29,30,31]. Software packages are available for comparison of these sequences with those of candidate ligands, an approach that has led to the identification of binding sites on a number of proteins [32,33,34]. In these phage-display experiments, random nucleotides are inserted into gene III of the M13 phage at a position that results in a peptide insert displayed on the surface of the virion. Each virion produced has a different peptide insert on its surface. An aliquot of this library is incubated above a target molecule (for instance, an integrin) and then iteratively washed, released and amplified to isolate those virions displaying peptides with the greatest affinity for the target. Most peptides selected for binding to RGD-binding integrins do, in fact, include an RGD motif. Since the reported peptides were from 6 to 15 amino acids in length, their binding to the target integrin was potentially modulated by the adjoining non-RGD amino acids. For instance, sequences of peptides selected for binding to integrin α_1_β_5_ should exhibit the greatest similarity to sequences of ligands known to bind integrin α_1_β_5_. Similarity of short peptide sequences to protein sequences is computed using the program MATCH [33] with a similarity matrix modified from BLOSUM62 [35] for use with short sequences. An example of the alignment of peptides selected for binding to integrin α_v_β_3_ with the sequences of fibronectin and CoV-2 spike protein is shown in Figure 4. The peptides exhibit comparable similarity to the sequences of both of these proteins, consistent with the possibility that the RGD motif from the spike protein may exhibit an affinity for integrin α_v_β_3_.

Sequences of peptides selected for affinity to integrins α_iib_β_3_, α_v_β_3_, α_v_β_5_ and α_1_β_5_ were compared to sequences of integrin ligands of known specificity to determine if the similarity was predictive of ligand–integrin specificity. For instance, 67% of the sequences of decapeptides selected for binding to integrin α_v_β_5_ exhibited similarity to the sequence of osteopontin, a protein known to bind to integrin α_v_β_5_, whereas only 7% of these peptides exhibited similarity to the sequence of thrombospondin, a protein that does not bind integrin α_v_β_5_. When at least 20% of selected peptides exhibited similarity to the integrin ligand, the screen proved predictive. Using this criterion, 80% of the screens predicted the correct integrin specificity for the ligand.

Table 2 documents the proportion of selected peptides that exhibit similarity to nine integrin ligands of known specificity. Examination of this data indicates that some libraries have better predictive power than others. The predictive power of each library is defined as the percentage of integrin ligand specificities that the library correctly predicted. Five of the libraries correctly predicted binding for at least two thirds of the nine integrin ligands queried.

The available data are derived from libraries that used different scaffolds and topologies of peptide display, precluding quantitative comparisons. Nevertheless, patterns of similarity are apparent when the sequences of peptides selected for binding to specific integrins are compared to those of integrin ligands with known specificity. On this basis, the CoV-2 spike protein is a candidate for binding to integrins α_v_β_3_ and α_v_β_5_. The available data are not sufficiently predictive to make any statement about the possibility of CoV-2 spike protein binding to other integrins.

## 3. Vulnerability of the Integrin Signaling Network

**Figure 5:**
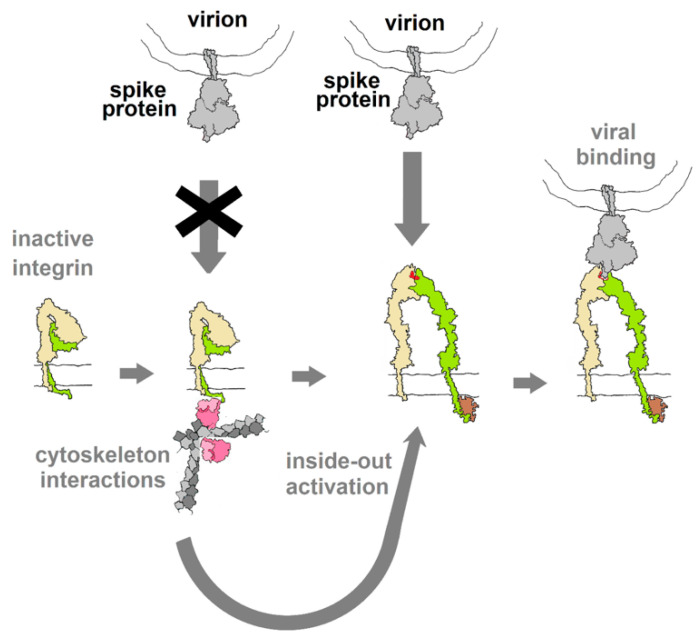
Diagram of the hypothesized activation of integrin through interactions with elements of the cytoskeleton (inside-out activation), as a result of other cellular stimuli (outside-in). This switches the integrin into a conformation with affinity for RGD motif ligands. If binding of spike protein mimics the action of integrin ligands (acts as an agonist), this binding may be capable of activating downstream cellular activities. Integrin drawing based on PDB Molecule of the Month (https://pdb101.rcsb.org/motm/134).

Integrins play critical roles in cellular adhesion, cell–extracellular matrix (ECM) interactions and signaling networks that are vital aspects of many cellular functions [36] and, as such, represent a gaping vulnerability in human physiology that numerous viruses exploit. The vulnerability arises from the extracellular binding sites that recognize the RGD motif among other structural cues. Normally, in an inactive state with low affinity for natural ligands, integrins are activated through interactions with elements of the cytoskeleton, triggering a conformational change that raises their affinity to ligands and makes them susceptible to binding by viral proteins that may dysregulate integrin-mediated processes, as diagrammed in Figure 5. Virus particles that display “RGD” on their surface invariably utilize integrins as sites of attachment and cell entry and, in many cases, also trigger signal pathways that control aspects of cellular development, growth and motility [18].

Integrins are obligate heterodimers composed of an α-chain and β-chain. The human proteome contains 18 α-chains and 8 β-chains that can combine to form 24 functionally distinct integrins [37,38]. They have large extracellular domains, a single trans-membrane helix and (in most cases) a small cytoplasmic domain. At least eight are known to bind ligands displaying the RGD tri-peptide motif [37] in a cleft between the α- and β-domains [36,37]. Virions displaying RGD motifs also bind at these sites.

The use of a tri-peptide motif as a recognition signal to regulate such a wide array of cellular and tissue processes cannot, in and of itself, provide adequately precise control of cellular processes. Over 7% (1593) of the 20,619 protein sequences listed in the most recent uniprot human proteome (https://www.uniprot.org/proteomes/UP000005640) contain RGD motifs. The vast majority of these do not participate in integrin-mediated signaling. Most are proteins confined to the cytoplasm of cells, sequestered from the extracellular integrin-binding sites. Even in extracellular proteins, the RGD motifs may be conformationally locked, integrated into the protein structure in a manner that precludes integrin binding. Analysis of the conformation of the spike protein RGD motif (detailed above) suggests that, although the motif is not fully exposed, the surface loops surrounding it are sufficiently flexible that interactions with integrins may be possible.

### Diverse Forms of Virion–Integrin Interactions

**Figure 6:**
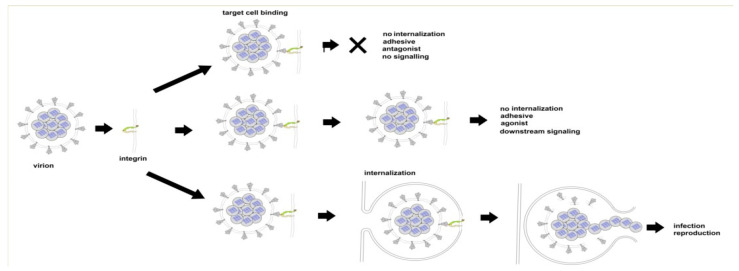
Three modes of viral interaction with cell surface integrins. Top: Binding without internalization or the triggering of integrin-mediated pathways (virion acting as antagonist). Middle: Binding without internalization but with the triggering of integrin-mediated pathways (virion acting as agonist). Bottom: viral internalization leading to infection and viral replication.

There are three ways in which the binding of SARS CoV-2 to an integrin could impact tissue integrity. The simplest (Figure 6 top) is for the virion to bind and act as an antagonist, a competitive inhibitor that blocks the interaction of the integrin with a ligand, disrupting signaling and downstream cellular responses due to that ligand. The second way (Figure 6, middle) is for the virion to act as an agonist, triggering signaling pathways and leading to dysregulated and potentially damaging cellular responses. The third way (Figure 6, bottom) is to utilize the integrin as a portal for cell entry, leading to viral replication and cellular disruption. In the first two cases, the virion may remain attached to the cell surface as a multivalent adhesive element that could contribute to uncontrolled cell–cell adhesion. If bound to platelets, this could contribute to platelet aggregation and the coagulopathies frequently observed in COVID-19.

## 4. Viral Entry via Integrins

Binding of the spike protein to the ACE2 receptor can initiate host cell recognition and viral entry for both SARS CoV-1 and SARS CoV-2 virions (Zhou et al., 2020). SARS CoV-2 appears to have 10–20× greater affinity for ACE2 than observed for SARS-CoV-1, perhaps due to increased flexibility in the spike protein receptor-binding domain [23,24]. This greater affinity has been implicated in the significantly greater infectivity of SARS CoV-2, a primary driver of the current pandemic. However, evidence reviewed above suggests that alternate pathways for entry of CoV-2 into cells may contribute to its very high infectivity and increased flexibility of the receptor-binding domain could increase exposure of the RGD motif, thereby enhancing integrin-binding.

**Figure 7:**
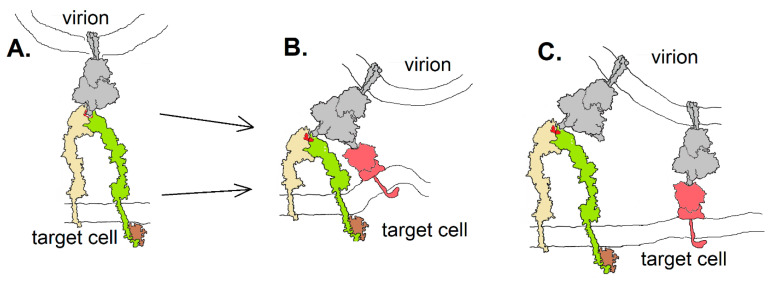
Three hypothetical modes of spike protein (gray) binding to integrin (yellow and green): (**A**) in an ACE2-independent way; (**B**) as a co-receptor in which a single spike protein interacts with both integrin and ACE2 (red) as facilitated by the flexibility of the receptor binding domains; (**C**) as a co-receptor in which multiple spike proteins interact with either an integrin or ACE2. Integrin drawing based on PDB Molecule of the Month (https://pdb101.rcsb.org/motm/134).

Integrins could contribute to SARS-CoV-2 entry into host cells in at least three different ways (Figure 7): they could act as an ACE2-independent route to cell entry (Figure 7A), providing viral access to cells that do not express ACE2; they could act as co-receptors, enhancing the affinity of the virion to a target cell by either binding to the same spike protein as an ACE2 receptor via a second receptor binding domain or by involving additional spike proteins in cell attachment. The “stalk” of the spike protein has three hinges [39], providing it with great flexibility to orient in a manner required to bind a receptor—be it ACE2 or an integrin. The receptor-binding domains are connected to the remainder of the protein by a highly flexible link, able to orient in an “open” or “closed” configuration [24]. Nevertheless, it is unknown whether this flexibility would be adequate to accommodate binding of the spike protein to two receptors simultaneously, as in the hypothetical drawing in Figure 7B. If integrins act as co-receptors for CoV-2, it may be less sterically restrictive to do so by involvement of multiple spike proteins, as diagrammed in Figure 7C.

Integrins are used as cell-entry portals by many human viruses. Adenovirus utilizes an initial recognition step followed by interaction of an RGD motif in its “penton base” proteins with integrin α_v_β_3_ [40] and perhaps α_v_β_5_ and α_5_β [41] to mediate cell entry. Binding of the penton to α_v_β_3_ integrins promotes integrin clustering and subsequent internalization [42]. Whether this clustering is due to cross-linking by the pentavalent penton base, or to the penton proteins acting as agonists to trigger integrin-mediated signaling, is not clear.

Foot and mouth disease virus (FMDV) utilizes RGD-binding to four integrins (α_v_β_1_, α_v_β_3_, α_v_β_6_ and α_v_β_8_) for attachment and subsequent entry via clathrin-coated pits [43]. Human herpes viruses, including Kaposi’s Sarcoma Herpesvirus (KSHV), Cytomegalovirus (CMV), Herpes Simplex Virus (HSV) and Epstein–Barr virus (EBV), all appear to use RGD motifs displayed on virion glycoproteins to gain cell entry [44] through binding to integrins α_3_β_1_, α_V_β_3_ and α_V_β_5_ [45]. Given the frequent use of integrins for cell entry by other viruses, it would not be surprising to find that SARS CoV-2 also uses integrins as receptors, secondary receptors or co-receptors.

Should SARS CoV-2 use integrins as a way to supplement ACE2-mediated cell entry, integrins could provide a path for virion entry into cells, such as neurons, that do not express ACE2 at high levels.

## 5. Integrins, SARS CoV-2 and Coagulation

COVID-19-associated coagulopathy is multifactorial, involving venous, arterial and microcirculatory systems in a way that appears distinct from other viral illnesses (Becker, 2020). Autopsies have revealed unique pathological features of COVID-19 including diffuse small vessel (venule, arteriole and capillary) platelet–fibrin thrombosis and intravascular megakaryocytes in all major organs, including the heart, lungs, kidneys, liver and mesenteric fat [4]. In addition, this pathological coagulation may underpin one of the most devastating complications of COVID-19—pulmonary embolism, acute large vessel occlusion with ischemic stroke in patients less than 50 years of age [46]. These aspects appear to differentiate COVID-19-associated coagulopathy from other viral or bacterial-induced coagulation disorders. COVID-19-associated coagulopathy is distinct from disseminated intravascular coagulation often associated with sepsis and many other pathologies, in presenting an increased risk of thrombosis without bleeding [47]. Investigators have, to date, hypothesized that an imbalance between coagulation and inflammation may explain the observed hypercoagulable state [48].

Clotting in the vasculature is commonly initiated by the disruption or activation of the endothelial cells lining the blood vessels that normally have an anticoagulant function because of the thrombomodulin-protein C system. In hemostasis, clotting is initiated by the disruption of the endothelium and expression of tissue factor, exposure of collagen or other subendothelial components. In thrombosis, clotting is commonly initiated by reactive oxygen species that disturb the endothelium or ruptured atherosclerotic plaque. There is now considerable evidence that SARS-CoV-2 virions bind endothelial cells and enter these cells by an ACE2-dependent and/or ACE2-independent pathway. This process stimulates endothelial signaling pathways as described above and activates the endothelial cells, leading to activation of platelets and the coagulation pathway, initiating the intense coagulopathy observed in COVID-19 patients.

With viral invasion, the activated endothelium would cause platelet adhesion and activation with coincident switching of integrin α_IIb_β_3_ into a high-affinity conformation [49]. Fibrinogen binds to activated integrin α_IIb_β_3_ and is quickly converted to fibrin by the proteolytic activity of thrombin on the platelet surface. Platelet aggregation is then driven by binding of monomeric, oligomeric and polymeric fibrins to integrin α_IIb_β_3_ molecules on adjacent platelets, linking them to one another in a rapidly growing clot [49].

**Figure 8:**
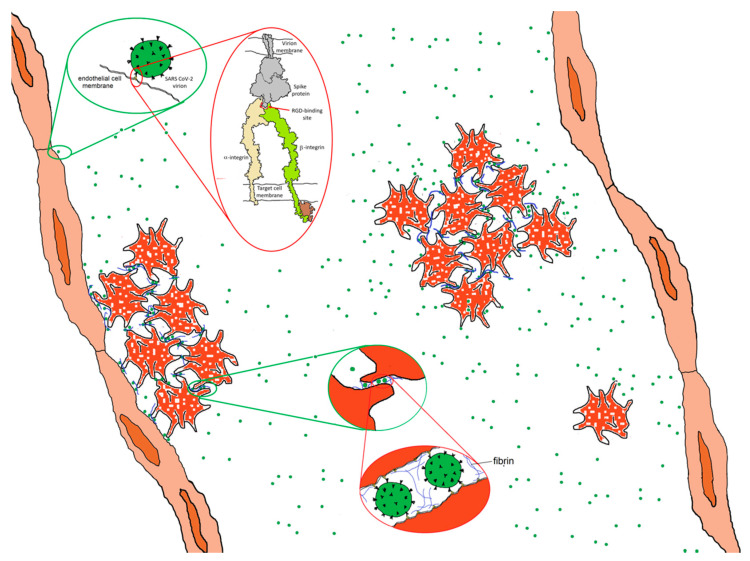
Hypothetical adhesive impact of the interaction of SARS CoV-2 spike protein to integrins. Spike protein interacting with an activated integrin molecule (top inset) provides a mechanism for adhering to endothelial cells and, potentially, platelets, contributing with fibrin to platelet adhesiveness and aggregation (bottom inset). Integrin drawing based on PDB Molecule of the Month: https://pdb101.rcsb.org/motm/134.

It is also possible that direct virion–platelet interactions are involved [50]. Platelets express several integrins including α_IIb_β_3_, α_v_β_3_, α_2_β_1_, α_5_β_1_ and α_6_β_1_ [51]. α_IIb_β_3_ is expressed at far higher levels than the others and plays a key role in clotting, acting as a membrane-anchored cleat to secure fibrinogen and fibrin fibers to the platelet surface so as to aggregate platelets into mechanically robust clots. If the RGD motif on the SARS-CoV-2 virion exhibits affinity for a platelet integrin, it would have the potential to bind to platelets in a manner analogous to fibrin and thereby adhere platelets to one another, as shown schematically in Figure 8. There are of-the-order-of 100 spikes on the surface of SARS CoV-2, each one a trimer containing three RGD motifs. It requires little stretch of the imagination to realize that, when introduced into the bloodstream, virions displaying proteins that exhibit high affinity for platelet integrins in their active conformation could precipitate clot formation, giving rise to microthrombi and coagulopathy throughout the body. This strategy appears to be used by Hantaviruses [52]. Carried largely by rodents, Hantaviruses can cause a variety of serious illnesses. Although their virions do not appear to have an RGD motif, they bind β_3_ integrins nonetheless. Binding to integrin α_IIb_β_3_ or α_v_β_3_ on platelets and integrin α_v_β_3_ on endothelial cells [52], Hantavirus virions act as efficient adhesive elements to recruit platelets to endothelial cell surfaces, causing the build-up of thrombi on vascular walls [52]. Could a similar mechanism contribute to coagulopathies in COVID-19?

In the previous SARS-CoV-1 outbreak, thrombotic complications were reported [53] but were apparently less consequential than those observed in COVID-19 [54,55,56]. The SARS CoV-1 spike protein contains a KGD motif at a position homologous to that of the RGD motif in CoV-2. Depending on structural context, the KGD motif may exhibit affinity for integrins and has been used as a basis for the design of pharmaceuticals targeting integrin α_IIb_β_3_ [57]. Whether the KGD motif in SARS CoV-1 spike protein contributed to observed coagulopathies in SARS remains unclear. The MERS spike protein has no corresponding sequence, and no MERS-related coagulopathy appears to have been reported [55,56].

Should virion–integrin interactions prove to contribute to COVID-19 coagulopathies, they almost certainly act in concert with a hyper-inflammatory response [48]. Coagulopathies are common in severe viral or bacterial infections, where inflammation can promote coagulation by triggering the expression of intravascular tissue factor, leukocyte adhesion molecules on intravascular cell surfaces and downregulating fibrinolytic and other anticoagulant pathways [58]. Dysfunctional coagulation is a common complication in severe influenza [59], contributing to a high incidence of thrombotic complications, including deep venous thrombosis, pulmonary thrombosis and embolism, limb ischemia, ischemic stroke and myocardial infarction. The hypothesis put forward here is that coagulation induced by the inflammatory response to SARS CoV-2 is dramatically augmented by direct interactions of virions with endothelial cells and platelets.

## 6. Integrins, SARS CoV-2 and Angiogenesis

Although the induction of angiogenesis is most often associated with the action of vascular endothelial growth factor (VEGF), at least six integrins, α_V_β_3_, α_V_β_5_, α_5_β_1_, α_2_β_1_, α_V_β_1_ and α_1_β_1_, have been implicated as contributors [20,21]. If the spike protein of SARS CoV-2 exhibits affinity for integrins, it may be capable of triggering dysregulated angiogenesis. Intussusceptive angiogenesis, commonly observed in the lungs of COVID-19 victims on autopsy [3], is a dynamic intravascular process capable of modifying the structure of the microcirculation through the formation of cylindrical microstructures that span the lumen of small vessels and capillaries [60]. The disruption of pulmonary tissue resulting from uncontrolled angiogenesis contributes to the extensive lung damage observed in the most severe cases. For SARS CoV-2 virions to induce processes of this kind, the RGD motif on the spike protein would need to act as an agonist, activating, among others, integrins α_V_β_3_ and/or α_V_β_5_, long considered to be positive regulators of the angiogenic switch [61,62]. This is not unprecedented—activation of signaling pathways by the interaction of virion-displayed RGD motifs with integrins is relatively common in human viruses. For instance, Kaposi’s sarcoma-associated virus (KSHV) utilizes an RGD motif on surface glycoprotein gB to bind host cell integrins, gain cell entry [44] and act as an agonist to activate numerous signaling pathways [63]. KSHV triggers the association of integrins with Rho GTPases, tyrosine kinase receptors and Toll-like receptors that results in cytoskeletal remodeling, differential cell type targeting and innate responses [64]. These activities are mediated through at least three integrins [45]. It represents a prime example of how the interaction of RGD motifs with integrins can lead to virally induced activation of integrin-mediated pathways.

## 7. Discussion

### 7.1. Integrin Antagonists as Potential COVID-19 Therapies

Should virion–integrin interactions prove to be important in viral entry into the alveolar cells or the generation of thrombotic and angiogenic manifestations in COVID-19, pharmaceuticals designed to block or disrupt these interactions could mitigate some of the worst clinical features of COVID-19. A drug that blocked the binding of virions to endothelial cells or platelets could potentially slow virally induced clot formation. Similarly, a pharmaceutical that prevented the binding of virions to integrins implicated in angiogenesis could block downstream signaling required for angiogenesis, mitigating the associated tissue disruption and damage. The development of reagents targeting integrins has been pursued for decades [65] but has been hampered by the challenge of targeting specific integrins and by a lack of understanding of why some integrin-binding molecules act as antagonists while others act as agonists. As antagonists, they can block binding by natural ligands, shutting down signaling pathways; as agonists, they are capable of mimicking natural ligands, inducing signaling and amplifying (rather than suppressing) the targeted pathogenesis.

Anti-thrombotic drugs targeting integrin α_IIb_β_3_ have achieved only limited success. Theoretically, a molecule binding tightly to integrin α_IIb_β_3_ should act as a competitive inhibitor, blocking fibrinogen-binding and the consequent platelet aggregation and clotting. Some small-molecule mimics of RGD-containing ligands, contrary to design, appear to act as agonists, triggering platelet activation and thereby enhancing clotting rather than blocking it [65,66]. Under the right circumstances, biologics, such as antibodies, antibody fragments or cyclic peptides, appear to function as designed, acting as antagonists, binding integrin α_IIb_β_3_, blocking fibrinogen binding to platelets and suppressing thrombus formation. However, they require intravenous delivery, which has limited their use [57,65]. Nevertheless, their potential use for the treatment of late-stage COVID-19 should be explored if SARS CoV-2 virions are shown to bind integrins.

There are many other integrin-targeting drugs, some especially for lung inflammation, including those for pulmonary fibrosis, which could be considered promising. Molecules targeting integrins implicated in angiogenesis have the potential to block virion-binding, preventing the signaling that appears to be an obligate step in virion-induced angiogenesis. Although the degree to which integrins such as α_v_β_3_ and α_5_β_1_ are expressed on the interior surface of COVID-19-infected pulmonary vasculature is unknown, if the hypothesized interactions between virions and integrins are drivers for the observed lung damage including dysregulated angiogenesis, integrins must be present on relevant vascular surfaces. Several antagonists of integrins α_5_β_1_ and α_v_β_3_ have been tested as anti-angiogenics [67]. If SARS CoV-2 virions bind to integrins, the potential utility of these pharmaceuticals in preventing COVID-19-related tissue damage should be investigated.

Two reports of patients being treated for multiple sclerosis with an integrin antagonist, natalizumab, which acts by binding to α_4_ integrins, have reported favorable outcomes [68,69]. These results led Aguirre et al. [68] to speculate that natalizumab might have potential as an antiviral drug against COVID-19. Natalizumab appears to act as an integrin antagonist and, as such, its potential as an antiviral for CoV-2 is supported by the arguments put forward here.

### 7.2. The Peril of Future Coronavirus Evolution

**Figure 9:**
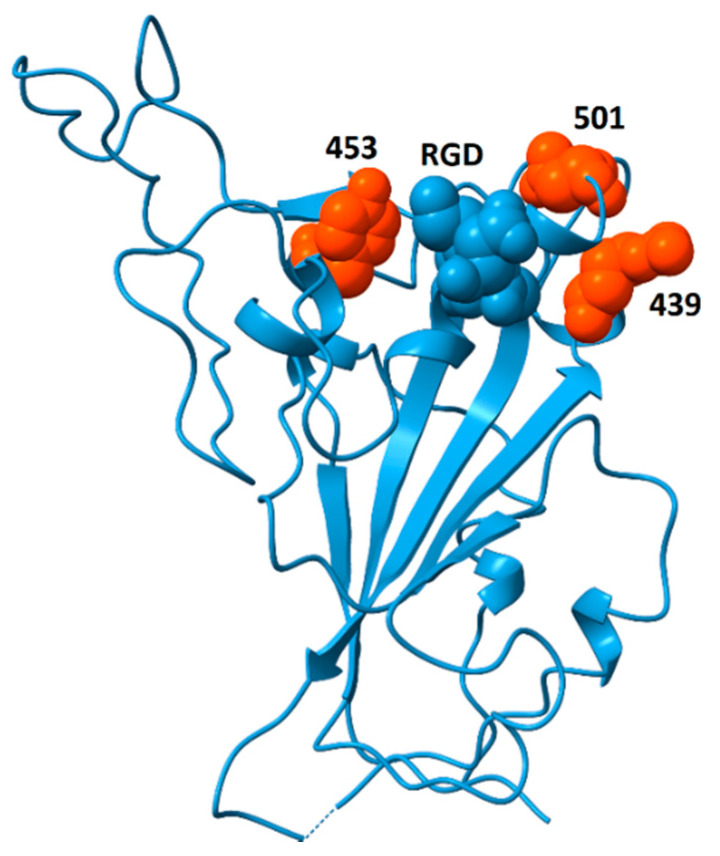
Spike protein receptor binding domain showing the location of residues N439, Y453 and N501 (orange) surrounding the RGD motif (blue, space-fill). The double mutant Δ69/Δ70 often co-occurs with single-amino-acid replacements at these locations (N501Y, N439K and Y453F). Although residues 439 and 501 participate in ACE2-binding, Y453 does not [71]. This model is from pdb file 6M0J, in which the receptor-binding domain is bound to the ACE2 receptor (receptor not shown). Structural models in which the receptor-binding domain is unbound generally do not contain coordinates for residue 501 due to flexibility of the peptide chain between residues 499 and 504. The strategic location of this residue suggests that substitutions at this site could impact the availability of the RGD motif for binding to integrins.

Analysis of genomic features of coronaviruses [70] identified two dominant factors associated with the emergence of viruses with high case fatality rates: (i) nuclear localization signals in the nucleoprotein and (ii) insertions or deletions in the spike protein sequence that lead to increased flexibility, resulting in higher affinity for ACE2 and, perhaps, other surface receptors, thereby enhancing infectivity. The analysis of spike protein structure presented here considers the potential for spike protein binding to integrins and discusses the pathogenic impacts that may arise from those interactions. If increased flexibility of spike protein provides a competitive advantage through greater binding affinity to ACE2, a side effect of this evolutionary pressure could be increased solvent exposure of the RGD motif. If this were to happen, it could generate coronavirus strains with increased affinity for integrins and enhanced pathological features including increased ability to zoonotically transmit to humans; acceleration of cell entry and infectivity; and increased triggering of integrin-mediated pathways, resulting in disease with additional extrapulmonary manifestations. Recently, a highly infectious variant, B.1.1.7 (or VUI 202012/01), of CoV-2 has been reported in the United Kingdom (Rambout, et al., 2020; Wise, 2020). The variant is characterized by multiple mutations including three amino acid deletions (69–70 and 144) and seven single-amino-acid replacements in the spike protein (N501Y, A570D, D614G, P681H, T716I, S692A, D1118H). Of these sequence changes, only N501Y is located in the receptor-binding domain. Residue 501 sits on the ACE2-binding surface [71] and this substitution appears to increase affinity to ACE2 [72,73], potentially explaining the greater infectivity. A side effect of this replacement may be greater surface exposure of the RGD motif. N501 is located immediately distal to the RGD motif and replacement of asparagine by tyrosine at that position could enhance the accessibility of the RGD motif for integrin-binding. Further support for this hypothesis comes from the observation that the double deletion mutant Δ69–Δ70 often co-occurs with the replacements N501Y, N439K and Y453F [74]. These replacements may increase ACE2-binding affinity [73], and their locations, bracketing the RGD motif, as shown in Figure 9, are well situated to modulate accessibility of the RGD motif for integrin-binding, potentially providing an additional competitive advantage.

## 8. Conclusions

The presence of an RGD motif in the spike protein of SARS CoV-2 combined with the observation of multiple clinical manifestations potentially linked to integrin function in COVID-19 suggests that virion–integrin interactions may contribute to viral entry, coagulopathy and dangerous tissue damage during the acute phase of disease. Other human viruses act through integrin-binding mechanisms to produce related symptoms. The spike protein of SARS CoV-2 contains an RGD motif that is missing in other coronaviruses and could underlie the molecular processes contributing to the observed coagulopathy and pathological angiogenesis. This motif has solvent accessibility similar to that of known integrin-binding ligands and a conformation consistent with the possibility of binding to integrins. Should this be the case, binding of intact virions to integrins could enhance viral entry into alveolar cells. Binding of virions to endothelial cells in the vasculature could lead to their activation and initiate blood clotting, contributing to the frequently observed widespread clotting and thrombosis in multiple organs. Furthermore, if virions bind to integrins on platelets, they could cause platelets to aggregate and bind to the endothelium. These processes would lead to the production of micro- and macro-thrombi in the vasculature, where they could pose a serious threat of pulmonary thrombosis and embolism, strokes and other thrombotic consequences. Binding of SARS-CoV-2 to endothelial cells could trigger angiogenic cell signaling pathways, leading to pathogenic intussusceptive processes with potential for amplifying tissue damage, and activation of other integrin-mediated cellular signaling pathways, leading to dysregulation of developmental processes and amplifying the impact of viral infection on multiple organs. The susceptibility of different tissues to these impacts may be modulated by a host of factors, including the conformation of relevant integrins and the state of the tissue microenvironment. Patient-specific differences in these factors may contribute to the highly variable clinical presentation of COVID-19. The emergence of the highly infectious variant, B.1.1.7 (or VUI 202012/01), which includes a receptor-binding domain amino acid replacement, N501Y, directly adjacent to the RGD motif, highlights the possibility that further viral evolution could generate variants with enhanced virion–integrin interactions and expanded biological and clinical impact.

## Figures and Tables

**Table 1:** Alignment of sequences in the neighborhoods of RGD motifs of CoV-1 and CoV-2 spike proteins and 12 known integrin ligands. Positively charged amino acids in red; negatively charged in green; hydrophobic in yellow.

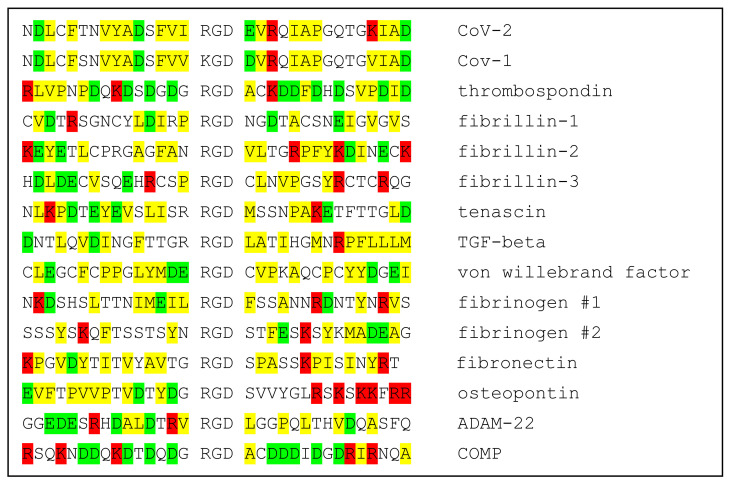

**Table 2:** A re-analysis of phage display data from experiments in which combinatorial phage-displayed peptide libraries were screened for affinity to four different integrins. Peptide sequences were reported by publications dating from 1992 to 2003. The first column indicates the integrin target for the affinity screen. The second column indicates the format of the peptide. Those peptides bracketed by cysteines, “c”, were assumed to be cyclic due to the predicted formation of disulfide bonds between the first and last displayed amino acid. “x” indicates a position where a random amino acid was inserted. In some cases, specific amino acids were inserted at defined positions within the peptide, and those are indicated according to the one-letter amino acid code. In column 3, the # of unique peptide sequences reported is given, a number which varied dramatically from study to study. The next 9 columns indicate the percentage of peptides that exhibited similarity to the RGD motif region of each of 9 known integrin ligands. There are two columns for fibrinogen corresponding to the two RGD motifs in the alpha-chain. “Similarity” was judged by the program MATCH using default parameters [33]. Green shading indicates those integrins that bind each ligand. For instance, osteopontin binds α_v_β_3_, α_v_β_5_ and α_5_β_1_, but not α_iib_β_3_. Empirically, when 20% or more of the selected peptides exhibited similarity for the RGD motif of an integrin ligand, it indicated that the ligand was likely to bind that integrin. The 12th column is predictive power defined as the percentage of correct predictions of integrin specificity made by each of the 10 library screenings. Yellow shading indicates a “predictive power” of 2/3 or greater. The next two columns are the proportion of peptides exhibiting similarity to the RGD/KGD motifs in CoV-2 and CoV-1 spike proteins. Brown shading indicates those screens that resulted in greater than 20% of the peptides exhibiting similarity to the motif region. The last column is the citation for the publication that reported the peptide sequences.

Target	Library Format	# Peptides	Fibronectin	Osteopontin	Thrombospondin	Vitronectin	Fibrillin	Fibrinogen 1	Fibrinogen 2	Tgf-beta	vwf	Predictive Power	Cov-2	Cov-1	Reference
α_iib_β_3_	cxxxxxxc	8	0	0	0.12	0.37	0	0.25	0.12	0.25	0	0.55	0	0.12	O’Neil et al 1992
α_iib_β_3_	xxxrgdxxxx	16	0.43	0.31	0.19	0.75	0.87	0.06	0.31	0.62	0.12	0.33	0	0	Li et al 2003
α_v_β_3_	xqxxxxxxsx	9	0.78	0.67	0.33	0.22	0.11	0	0.33	0.22	0.22	0.78	0.33	0.11	Li et al 2003
α_v_β_3_	xxrgdxxxxxx/xxxrgdxxxxx	28	0.75	0.61	0.39	0.57	0.21	0.04	0.64	0.07	0.46	0.78	0.28	0.11	Li et al 2003
α_v_β_3_	xxxxxx	45	0.22	0.31	0.2	0.31	0.09	0.11	0.29	0.11	0.2	0.66	0.18	0.02	Healy et al, 1995
α_v_β_3_	xxxxxxxxxxxxxxx	20	0.3	0.45	0.4	0.35	0.4	0.35	0.4	0.25	0.4	1	0.35	0.1	Healy et al, 1995
α_v_β_5_	xxrgdxxxxx	15	0.53	0.67	0.07	0.87	0.07	0	0.8	0.27	0.13	0.77	0.6	0.27	Li et al 2003
α_5_β_1_	xxxxxx	29	0.1	0.03	0	0.1	0.03	0.1	0.07	0.62	0.07	0.44	0.03	0	Koivunen et al 1993
α_5_β_1_	xxxxxxxxxxxxxxx	10	0	0.1	0	0.3	0.2	0.5	0	0.7	0.3	0.22	0.2	0	Healy et al, 1995
α_5_β_1_	cxxxxxxxc	75	0.17	0.12	0.16	0.28	0.07	0.16	0.15	0.08	0.11	0.44	0.1	0.04	Koivunen et al 1994
